# Universal health coverage: time to deliver on political promises

**DOI:** 10.2471/BLT.20.250597

**Published:** 2020-02-01

**Authors:** Viroj Tangcharoensathien, Anne Mills, Walaiporn Patcharanarumol, Woranan Witthayapipopsakul

**Affiliations:** a International Health Policy Program, Ministry of Public Health, Nonthaburi, 11000, Thailand.; b London School of Hygiene and Tropical Medicine, University of London, London, England.

The United Nations General Assembly Political Declaration of the High-Level Meeting on Universal Health Coverage (UHC)^1^ confirmed the Member States’ commitment to accelerate progress towards achieving UHC. However, challenges remain on how these commitments can be translated into actions. 

Various papers in this theme issue address technical and operational dimensions of UHC implementation: pooling of resources to increase risk sharing;^2^ improving governance of UHC through voice and accountability in UHC legislation;^3^ understanding the political economy of tax-financed systems;^4^ the roles of immunization and human immunodeficiency virus programmes in relation to UHC;^5^^,^^6^ the health workforce;^7^ and resource-tracking tools.^8^ Although the growing volume of scientific evidence contributes to reducing the knowledge gap in relation to UHC, the challenge of stimulating collective actions to move closer to this goal remains.

Moving towards UHC is a political decision. Evidence from several countries that have achieved UHC^9^^–^^11^ shows that policy entrepreneurs,^12^ who are strategically located in the policy network^13^ and in relatively stable institutional environments, are able to mobilize allies and overcome opposition to shape or achieve a specific policy agenda.^14^ In most cases, these policy entrepreneurs take advantage of the opening of policy windows,^15^ such as in a crisis context or in a major electoral and political realignment, to promote particular solutions.^14^^,^^16^

In the case of China’s reforms towards UHC, barriers to accessing health care and very high out-of-pocket costs have shifted the development agenda from economic growth to social harmony, including improving people’s livelihoods through UHC.^10^ The favourable fiscal space from sustained and high economic growth has supported UHC in the country. 

Thailand’s UHC policy was included in the political manifesto of the 2001 general elections. The country was able to achieve UHC one year later by abolishing voluntary health insurance and making insurance coverage a citizen entitlement. This achievement took place despite the country’s weak economy due to the 1997 Asian financial crisis. 

In Mexico, more than half of the population was uninsured in the early 2000s.^11^ This situation triggered a major reform towards UHC.

The experiences from these countries show that the efforts of internal policy actors are important for the achievement of UHC. Domestic capacities are critical in both shaping and implementing UHC.

The 2019 global UHC monitoring report^17^ uses two global monitoring indicators of the health-related sustainable development goal, UHC coverage index and incidence of catastrophic health expenditure at thresholds of 10% and 25% of household consumption. Globally, the service coverage index has improved from 45 out of 100 in 2000 to 66 out of 100 in 2017, but countries in conflict and fragile settings generally lag behind. The proportion of households that experience catastrophic health expenditure has increased from an average of 9.4% globally in 2000 to 12.7% in 2015. The African Region has greater challenges in monitoring financial risk protection due to limited data, and most fragile states and countries in conflict do not have these data.

Policy entrepreneurs need evidence to develop specific interventions to fill the UHC implementation gaps. Countries can be categorized in four groups by global averages of coverage and catastrophic expenditure, reflecting different policy needs.

First, countries in the high coverage and low incidence of catastrophic expenditure quadrant are good performers. Monitoring should focus on the sub-national level and on socioeconomic stratification and inequalities.

Second, countries with high coverage, but also high incidence of catastrophic expenditure, need to improve their financial risk protection through policies, such as more comprehensive benefit packages, inclusion of medicines and other cost–effective interventions, increased cost subsidies and the cessation of balanced billing.

Third, countries with low coverage and high incidence of catastrophic expenditure need to boost service provision and expand financial protection through, for instance, investing in essential infrastructure and, given fiscal constraints, prioritizing poor and vulnerable populations. Policy decisions should prioritize equity, since difficult policy choices may have to be made, between improving service capacity and expanding population coverage.

Finally, countries with low coverage and low incidence of catastrophic expenditure are the worst performers. Poor households that cannot afford health services may forego needed care. The magnitude and profiles of unmet health care needs should be assessed.^18^ Countries in this quadrant, often in complex emergency situations, need to accelerate progress on supply-side capacity and financial risk protection for the poorest and most vulnerable groups.

The Prince Mahidol Award Conference 2020 and UHC forum 2020 participants will discuss how to accelerate progress towards the political and operational dimensions of UHC.
